# *Larimichthys crocea*-Derived Calcium-Chelating Peptides Attenuate Leuprorelin-Induced Bone Loss and Lipid Metabolic Alterations in Mice

**DOI:** 10.3390/md24090301

**Published:** 2026-08-30

**Authors:** Xiaoping Wu, Shixian Zheng, Fang Liu, Junhong Liu, Yanyan Zhuang, Ding Li, Weiqing Huang

**Affiliations:** 1 College of Marine Sciences, Ningde Normal University, Ningde 352100, China; wuxiaoping1216@163.com (X.W.); zhengsx2004@163.com (S.Z.); lf_ndsy@163.com (F.L.); ljhxxx44@163.com (J.L.); zyy189zyy@163.com (Y.Z.); 2 Fujian Provincial University Engineering Research Center for Deep Processing of Mindong Aquatic Products, Ningde 352100, China

**Keywords:** *Larimichthys crocea*, calcium-chelating peptides, estrogen deficiency, bone loss, marrow adiposity, lipid metabolism

## Abstract

Estrogen deficiency induces bone loss and is accompanied by lipid metabolic disturbances. This study investigated the protective effects of *Larimichthys crocea*-derived calcium-chelating peptides (LCP) in a leuprorelin-induced estrogen-deficient mouse model. LCP attenuated tibial trabecular deterioration, increased serum procollagen type I N-terminal propeptide (PINP), osteocalcin (OCN), and bone-specific alkaline phosphatase (BALP), and reduced C-terminal telopeptide of type I collagen (CTX-I). Histological analyses further showed increased trabecular area and OCN expression and reduced marrow adiposity. LCP also attenuated alterations in serum lipid profiles and circulating levels of leptin and adiponectin, while reducing adipogenesis-related gene expression in femoral tissue and white adipose tissue. Exploratory assays using group-pooled cultures of bone marrow mesenchymal stem cells (BMSCs) showed differentiation-related patterns that were directionally consistent with the in vivo findings, but these descriptive observations require confirmation using independent biological replicates. Correlation analysis revealed associations among bone mass, osteogenesis-related indicators, and lipid metabolism-related parameters. Collectively, LCP attenuated estrogen deficiency-induced bone loss together with favorable changes in indicators related to bone formation, adipogenesis, and lipid metabolism. These findings support further investigation of LCP as a food-derived nutritional ingredient for supporting bone health under estrogen-deficient conditions.

## 1. Introduction

Osteoporosis is a systemic skeletal disorder characterized by reduced bone mass, deterioration of bone microarchitecture, and increased fracture risk [1]. Estrogen deficiency is one of the major causes of osteoporosis, particularly in postmenopausal women [2]. Decreased estrogen levels disrupt bone remodeling by impairing osteoblast-mediated bone formation and enhancing bone resorption, ultimately leading to trabecular deterioration and bone loss [2,3]. In addition to skeletal abnormalities, estrogen deficiency is closely associated with lipid metabolic disorders, including dyslipidemia, abnormal adipokine secretion, and excessive fat accumulation [3]. Increasing evidence indicates that bone and lipid metabolism are biologically interconnected, and estrogen deficiency is frequently accompanied by concurrent alterations in bone remodeling and lipid metabolism [1,4].

Bone marrow mesenchymal stem cells (BMSCs) are common progenitors of osteoblasts and adipocytes [5]. Under physiological conditions, the balanced differentiation of BMSCs toward osteogenic and adipogenic lineages is essential for maintaining bone homeostasis [1]. However, estrogen deficiency can shift BMSC lineage commitment away from osteogenesis and toward adipogenesis, resulting in reduced bone formation and increased marrow adiposity [3]. This imbalance is usually accompanied by changes in key osteogenic regulators, such as runt-related transcription factor 2 (*Runx2*) and *Sp7*, and adipogenic regulators, such as peroxisome proliferator-activated receptor gamma (*Pparg*) and CCAAT/enhancer-binding protein alpha (*Cebpa*) [1,2,3,6,7]. Therefore, maintaining a favorable balance between osteogenic and adipogenic differentiation may be an important strategy for protecting against estrogen deficiency-induced bone loss.

Marine organisms are valuable sources of structurally and functionally diverse bioactive compounds with potential nutritional and health-promoting applications [8]. In recent years, marine protein-derived bioactive peptides have attracted increasing attention because of their diverse biological activities and potential value in the development of functional ingredients [8,9]. Among these peptides, calcium-chelating peptides can bind calcium ions through specific amino acid residues and functional groups, which may improve calcium stability, solubility, and bioavailability [9]. Previous studies of food-derived calcium-chelating peptides have primarily demonstrated their ability to enhance calcium delivery, absorption, and utilization, as well as their potential to support osteogenic activity and bone formation. For example, herring egg phosphopeptides used as calcium carriers improved calcium absorption and bone microarchitecture in vivo [10], whereas a calcium-binding peptide–calcium chelate derived from bovine bone collagen hydrolysates exhibited pro-osteogenic activity associated with calcium-sensing receptor signaling [6]. Phosphorylated peptides from Antarctic krill have also been reported to attenuate bone loss and promote osteogenesis in a glucocorticoid-induced osteoporosis model [11]. In addition, our previous work showed that a fructooligosaccharide-*Larimichthys crocea* peptide copolymer enhanced calcium delivery and affected osteogenic and adipogenic differentiation-related outcomes in vitro [1].

Despite these advances, previous studies of food-derived calcium-binding or calcium-chelating peptides have largely focused on calcium bioavailability, osteogenic activity, and conventional bone-related outcomes [6,8,9,10,11]. Although beneficial skeletal effects of food-derived peptides have also been reported in ovariectomized estrogen-deficient models [2], the combined assessment of bone remodeling, marrow adiposity, systemic lipid metabolism, and BMSC differentiation has received comparatively limited attention. Accordingly, the present study evaluated *Larimichthys crocea*-derived calcium-chelating peptides (LCP) in a pharmacologically induced estrogen-deficient mouse model across these related skeletal and metabolic outcomes.

Leuprorelin is a gonadotropin-releasing hormone (GnRH) agonist that suppresses gonadal steroid production following prolonged administration [3]. Sustained GnRH receptor activation leads to pituitary desensitization, reduced gonadotropin secretion, and suppression of ovarian estrogen production. Previous studies have shown that GnRH agonist administration induces alterations in bone turnover and trabecular bone microarchitecture in rodents, supporting its use as a pharmacological model of estrogen deficiency-associated bone loss [3,12]. The present study aimed to determine whether LCP attenuates leuprorelin-induced bone loss and whether this effect is accompanied by changes in bone remodeling- and lipid metabolism-related parameters.

## 2. Results

### 2.1. Validation of the Estrogen-Deficient State and Effects of LCP on Tibial Trabecular Bone Microarchitecture

To verify the estrogen-deficient state induced by leuprorelin, serum estradiol (E2) levels were measured. Compared with the control group, serum E2 levels were significantly decreased in the model group (*p* < 0.05; Supplementary Figure S2), confirming the estrogen-deficient state following leuprorelin administration. The estradiol positive-control group (ES) showed significantly increased serum E2 levels compared with the model group, although the levels remained lower than those in the control group (*p* < 0.05). In contrast, no significant difference in serum E2 levels was observed between the LCP and model groups (*p* > 0.05).

**Figure 1:**
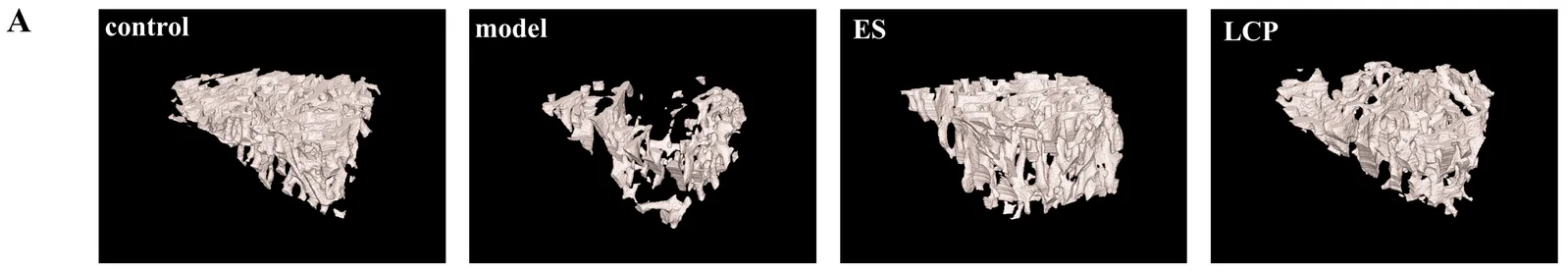

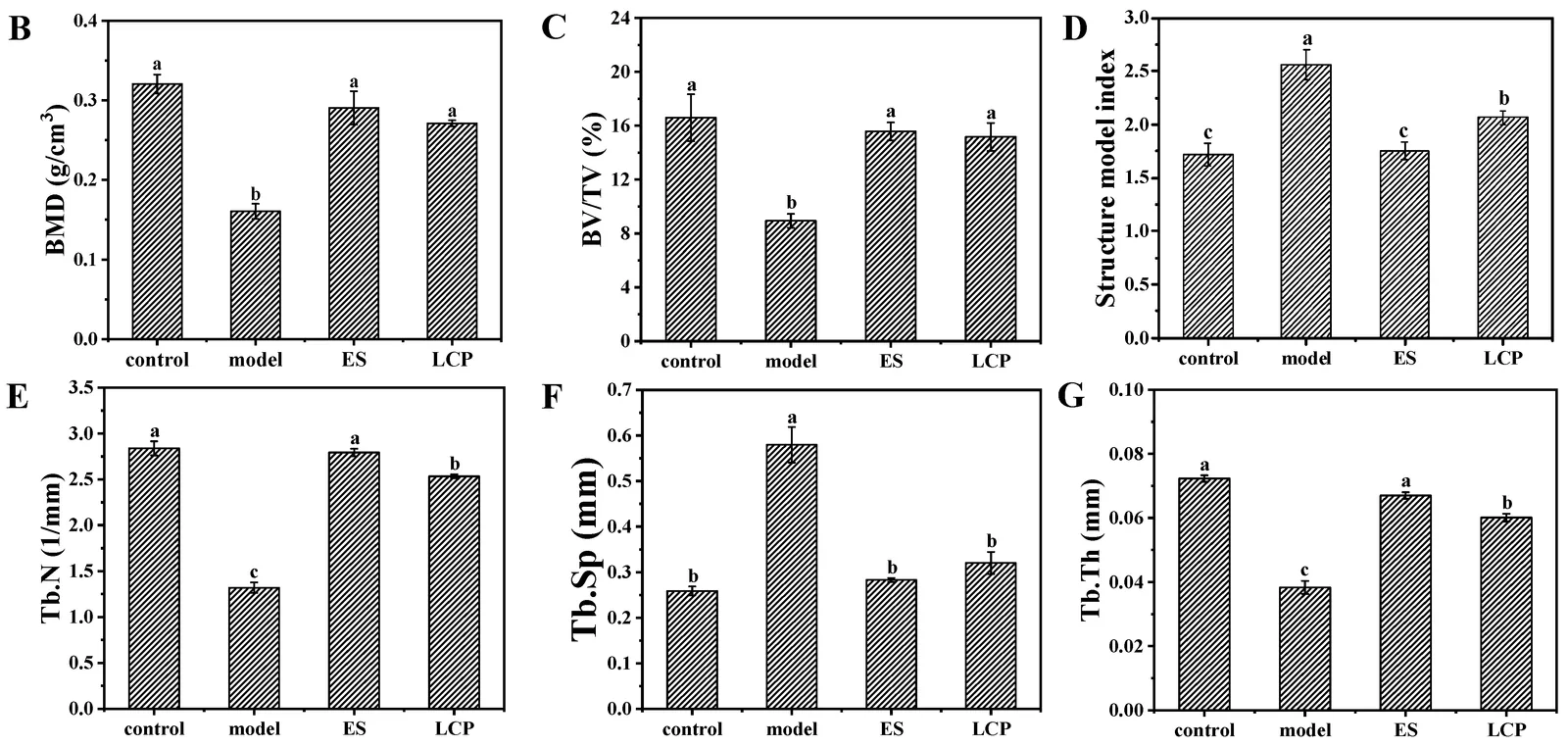
LCP attenuated deterioration of tibial trabecular bone microarchitecture in leuprorelin-induced estrogen-deficient mice: (**A**) Representative 3D micro-CT images of tibial trabecular bone. (**B**–**G**) Quantitative analysis of bone mineral density (BMD, (**B**)), bone volume fraction (BV/TV, (**C**)), structure model index (SMI, (**D**)), trabecular number (Tb.N, (**E**)), trabecular separation (Tb.Sp, (**F**)), and trabecular thickness (Tb.Th, (**G**)). Data are presented as mean ± SD (*n* = 6). Different lowercase letters indicate significant differences among groups (*p* < 0.05).

Micro-computed tomography (micro-CT) analysis was performed to evaluate the three-dimensional microarchitecture of tibial trabecular bone. Key structural parameters, including bone mineral density (BMD), bone volume fraction (BV/TV), structure model index (SMI), trabecular number (Tb.N), trabecular thickness (Tb.Th), and trabecular separation (Tb.Sp), were quantitatively analyzed. As shown in Figure 1A, the model group exhibited sparse and thin trabeculae with enlarged marrow spaces compared with the control group. Quantitative analysis further confirmed these structural changes. Compared with the control group, BMD, BV/TV, Tb.N, and Tb.Th in the model group were reduced by 49.90%, 46.17%, 53.52%, and 47.03%, respectively, whereas SMI and Tb.Sp were increased by 48.84% and 123.74%, respectively (*p* < 0.05; Figure 1B–G).

BMD and BV/TV in the ES and LCP groups did not differ significantly from those in the control group (*p* > 0.05; Figure 1B,C). SMI was significantly lower in both the ES and LCP groups than in the model group (*p* < 0.05; Figure 1D). SMI in the ES group was comparable to that in the control group, whereas the LCP group showed an intermediate value that remained significantly higher than those in the control and ES groups. In both the ES and LCP groups, Tb.N and Tb.Th were significantly increased, whereas Tb.Sp was significantly decreased compared with the model group (*p* < 0.05; Figure 1E–G). Although Tb.N and Tb.Th in the LCP group remained significantly lower than those in the control and ES groups, both parameters were significantly higher than those in the model group. Consistently, three-dimensional reconstructions showed denser trabecular networks in mice receiving either ES or LCP than in the model group. Collectively, these results indicated that concurrent LCP administration attenuated leuprorelin-induced deterioration of tibial trabecular bone microarchitecture.

### 2.2. Effects of LCP on Serum Bone Turnover Markers and Mineral Levels

**Figure 2:**
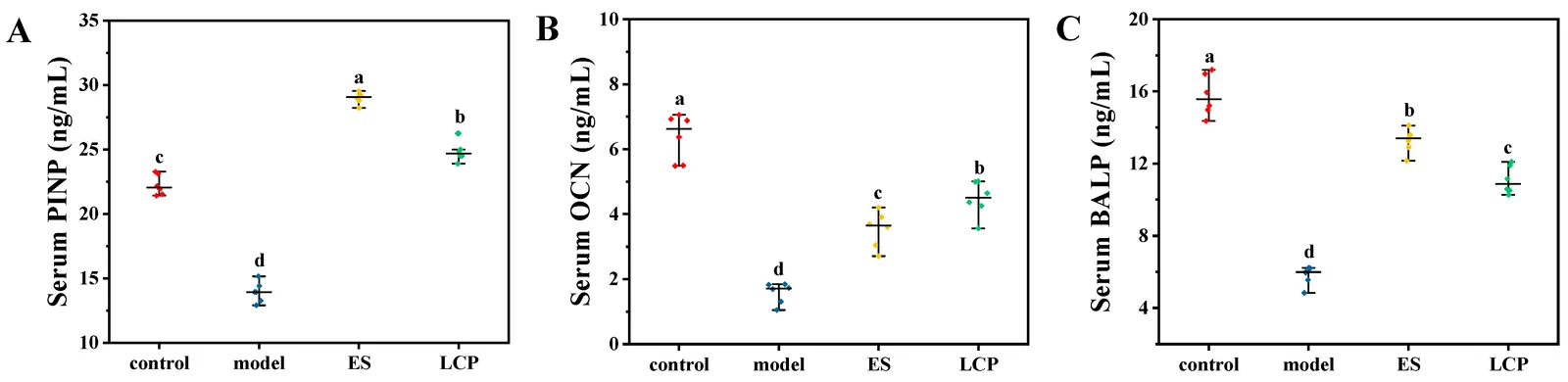

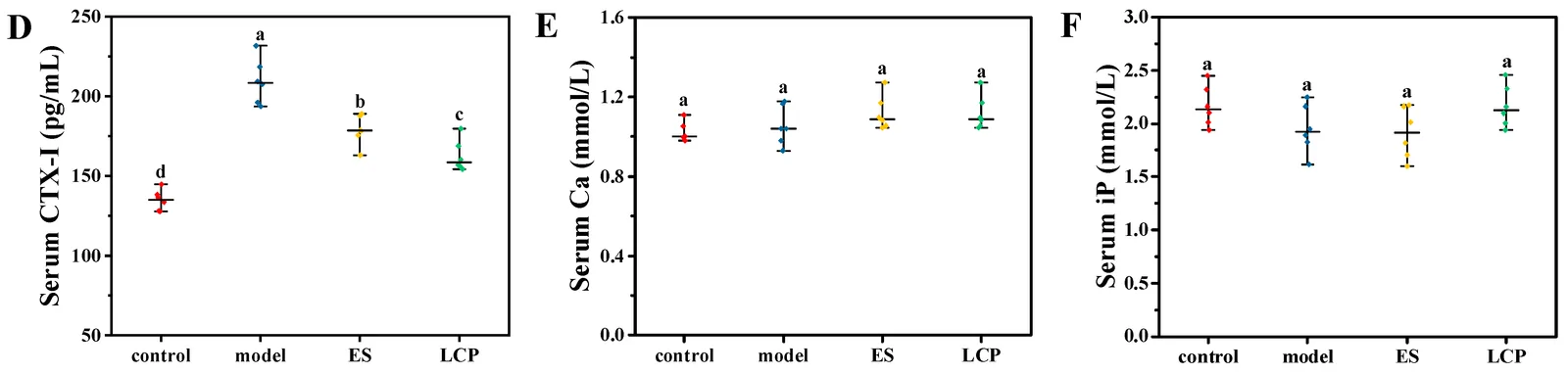
Effects of LCP on serum bone turnover markers and mineral levels. Serum levels of procollagen type I N-terminal propeptide (PINP, (**A**)), osteocalcin (OCN, (**B**)), bone-specific alkaline phosphatase (BALP, (**C**)), C-terminal telopeptide of type I collagen (CTX-I, (**D**)), calcium (Ca, (**E**)), and phosphorus (iP, (**F**)) were measured. Data are presented as mean ± SD (*n* = 6). Different lowercase letters indicate significant differences among groups (*p* < 0.05).

Serum bone formation markers, including procollagen type I N-terminal propeptide (PINP), osteocalcin (OCN), and bone-specific alkaline phosphatase (BALP), together with the bone resorption marker C-terminal telopeptide of type I collagen (CTX-I), were measured under leuprorelin-induced estrogen-deficient conditions (Figure 2A–D). Compared with the control group, the model group exhibited significantly decreased serum PINP, OCN, and BALP levels, accompanied by a significantly increased CTX-I level (*p* < 0.05). Compared with the model group, both the ES and LCP groups showed significantly higher serum PINP, OCN, and BALP levels and significantly lower CTX-I levels (*p* < 0.05). Serum calcium and phosphorus levels did not differ significantly among the groups (*p* > 0.05; Figure 2E,F). Collectively, concurrent LCP administration attenuated the alterations in serum bone turnover markers induced by leuprorelin without significantly affecting serum calcium or phosphorus levels.

### 2.3. Effects of LCP on Bone Remodeling- and Adipogenesis-Related Gene Expression in Femoral Bone Tissue

**Figure 3:**
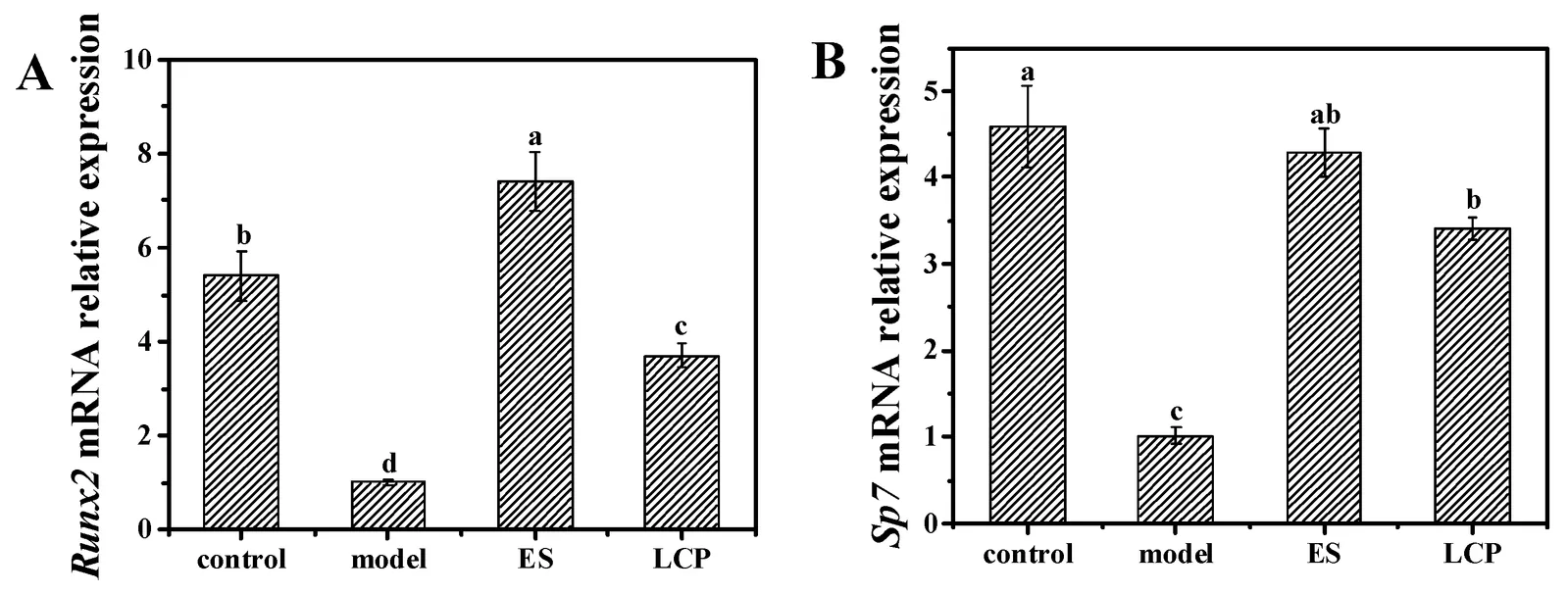

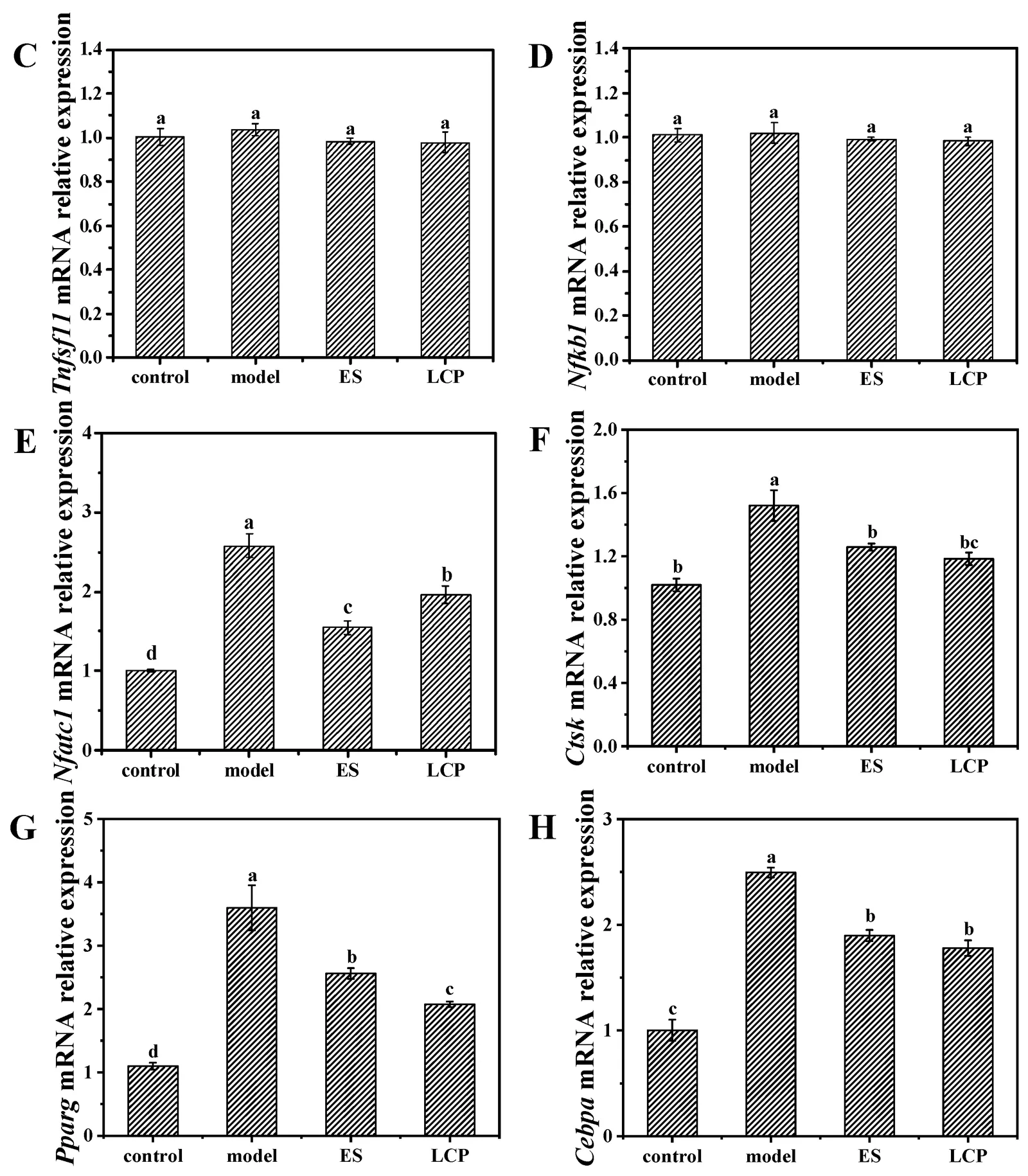
Effects of LCP on bone remodeling- and adipogenesis-related gene expression in femoral bone tissue. Relative mRNA expression levels of the osteogenesis-related genes *Runx2* (**A**) and *Sp7* (**B**), the osteoclastogenesis-related regulatory genes *Tnfsf11* (**C**) and *Nfkb1* (**D**), the osteoclast-associated genes *Nfatc1* (**E**) and *Ctsk* (**F**), and the adipogenesis-related genes *Pparg* (**G**) and *Cebpa* (**H**) were analyzed by RT-qPCR. Data are presented as mean ± SD (*n* = 6). Different lowercase letters indicate significant differences among groups (*p* < 0.05).

To further characterize transcriptional changes related to bone remodeling and adipogenesis, the relative mRNA expression of osteogenesis-related genes (*Runx2* and *Sp7*), osteoclastogenesis-related regulatory genes (*Tnfsf11* and *Nfkb1*), osteoclast-associated genes (*Nfatc1*, *Ctsk*, *Dcstamp*, and *Acp5*), and adipogenesis-related genes (*Pparg* and *Cebpa*) was examined in femoral bone tissue (Figure 3A–H and Supplementary Figure S3).

Compared with the control group, the model group exhibited significantly lower *Runx2* and *Sp7* expression (*p* < 0.05). The expression levels of both genes were significantly higher in the ES and LCP groups than in the model group (*p* < 0.05; Figure 3A,B). The expression levels of *Tnfsf11* and *Nfkb1* did not differ significantly among the groups (*p* > 0.05; Figure 3C,D). In contrast, the expression levels of *Nfatc1* and *Ctsk* were significantly increased in the model group compared with the control group and significantly decreased in both the ES and LCP groups compared with the model group (*p* < 0.05; Figure 3E,F). No significant differences in *Dcstamp* or *Acp5* expression were observed among the groups (*p* > 0.05; Supplementary Figure S3).

The adipogenesis-related genes *Pparg* and *Cebpa* were significantly upregulated in the model group compared with the control group (*p* < 0.05; Figure 3G,H). Compared with the model group, both the ES and LCP groups showed significantly lower expression of both genes (*p* < 0.05). Overall, compared with the model group, the LCP group showed higher expression of the osteogenesis-related genes *Runx2* and *Sp7*, lower expression of *Nfatc1*, *Ctsk*, *Pparg*, and *Cebpa*, and no significant differences in *Tnfsf11*, *Nfkb1*, *Dcstamp*, or *Acp5*.

### 2.4. Effects of LCP on Trabecular Structure, Marrow Adiposity, and OCN Immunoreactivity

**Figure 4:**
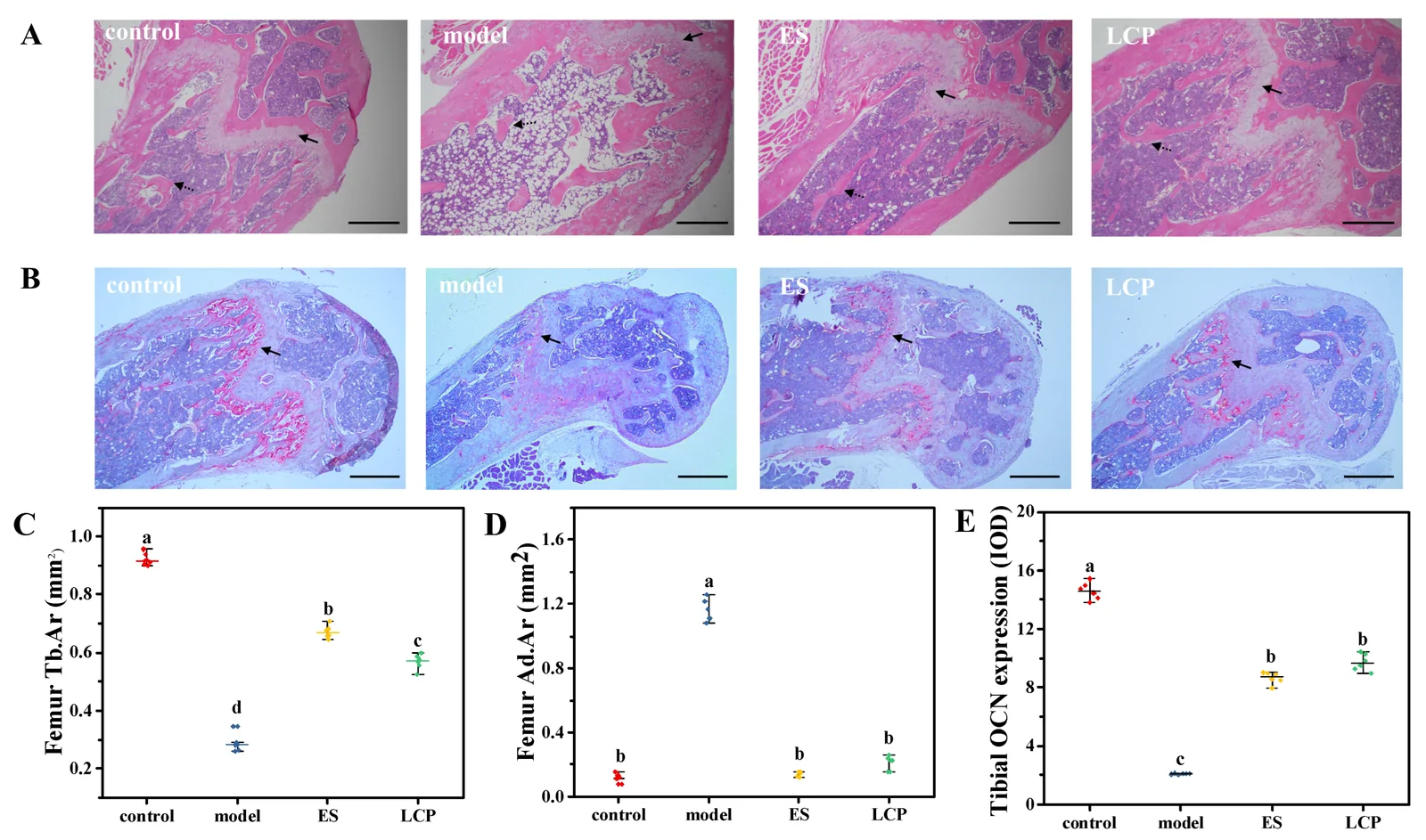
Effects of LCP on trabecular structure, marrow adiposity, and OCN immunoreactivity: (**A**) Representative H&E-stained femoral sections. (**B**) Representative OCN immunohistochemical staining of tibial sections. (**C**,**D**) Quantitative analysis of trabecular bone area (Tb.Ar, (**C**)) and marrow adipocyte area (Ad.Ar, (**D**)) in femoral sections. (**E**) Quantification of OCN-positive staining in tibial sections by integrated optical density (IOD). In (**A**), black dashed arrows indicate trabecular bone. Black solid arrows in (**A**,**B**) indicate regions adjacent to the growth plate, which were used as anatomical landmarks to ensure comparable image regions across groups. Scale bar: 100 μm. Data are presented as mean ± SD (*n* = 6). Different lowercase letters indicate significant differences among groups (*p* < 0.05).

Histological and immunohistochemical analyses were performed to assess trabecular morphology and marrow adiposity in femoral sections and OCN immunoreactivity in tibial sections, respectively. H&E staining of femoral metaphyseal sections revealed clear differences in trabecular morphology and marrow adiposity among the groups (Figure 4A). Compared with the control group, the model group exhibited sparser trabecular structures, enlarged marrow spaces, and abundant adipocyte-like vacuoles within the bone marrow cavity. In contrast, the ES and LCP groups showed better-preserved trabecular structures and fewer adipocyte-like vacuoles than the model group.

Quantitative analysis showed that trabecular bone area (Tb.Ar) was significantly decreased and marrow adipocyte area (Ad.Ar) was significantly increased in the model group compared with the control group (*p* < 0.05; Figure 4C,D). Both the ES and LCP groups showed significantly higher Tb.Ar and lower Ad.Ar than the model group (*p* < 0.05). Compared with the model group, the LCP group showed an 88.66% increase in Tb.Ar and an 86.83% decrease in Ad.Ar (*p* < 0.05). Although Tb.Ar in the LCP group remained significantly lower than that in the control and ES groups, Ad.Ar did not differ significantly from that in either the control or ES group.

OCN immunohistochemical staining was performed using tibial sections (Figure 4B). Compared with the control group, the model group showed markedly weaker OCN-positive staining and a significantly lower corresponding integrated optical density (IOD) (*p* < 0.05; Figure 4E). OCN-positive staining was more prominent in both the ES and LCP groups than in the model group, with significantly higher corresponding IOD values (*p* < 0.05). The IOD value in the LCP group was comparable to that in the ES group but remained significantly lower than that in the control group. Overall, compared with the model group, the LCP group showed a higher trabecular bone area, a lower marrow adipocyte area, and greater OCN immunoreactivity.

### 2.5. Body Weight, WAT Weight, and Lipid Metabolism-Related Outcomes in Estrogen-Deficient Mice

Body weight was monitored throughout the 12-week experimental period, and white adipose tissue (WAT) weight was recorded at the end of the study (Supplementary Table S1). No significant differences in body weight were observed among the groups at weeks 0, 4, 8, or 12 (*p* > 0.05). At the end of the experiment, WAT weight was significantly higher in the model group than in the control and ES groups (*p* < 0.05). Although the mean WAT weight in the LCP group was lower than that in the model group, the difference was not statistically significant (*p* > 0.05).

**Figure 5:**
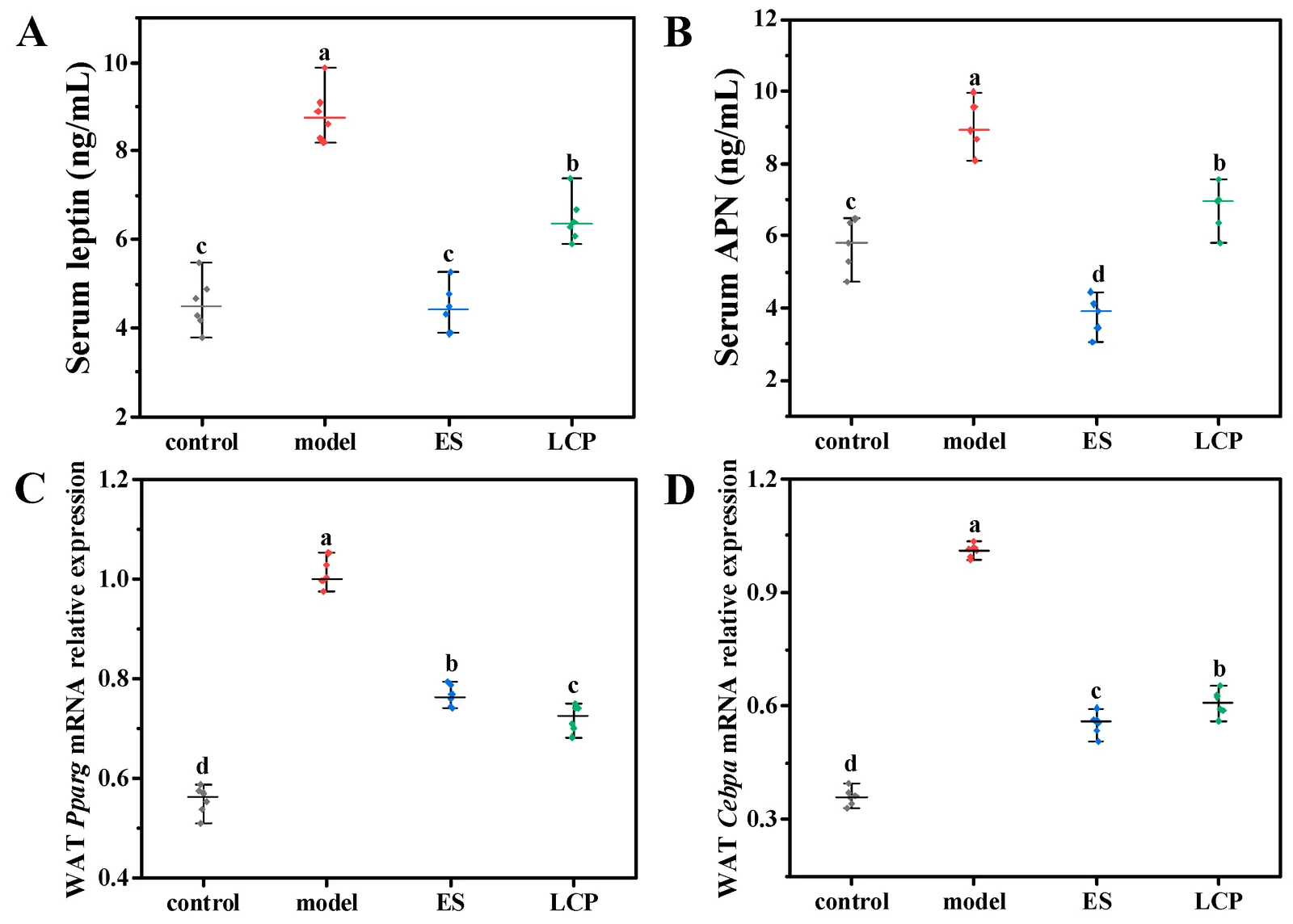
Effects of LCP on serum adipokines and adipogenesis-related gene expression in white adipose tissue (WAT) in leuprorelin-induced estrogen-deficient mice. Serum leptin (**A**) and adiponectin (APN, (**B**)) levels and relative mRNA expression of *Pparg* (**C**) and *Cebpa* (**D**) in WAT were measured. Data are presented as mean ± SD (*n* = 6). Different lowercase letters indicate significant differences among groups (*p* < 0.05).

Compared with the control group, the model group exhibited significantly higher serum total cholesterol (TC), triglyceride (TG), and low-density lipoprotein cholesterol (LDL-C) levels and a significantly lower high-density lipoprotein cholesterol (HDL-C) level (*p* < 0.05; Table 1). Compared with the model group, the LCP group showed significantly lower TC, TG, and LDL-C levels and a significantly higher HDL-C level (*p* < 0.05). Serum leptin and adiponectin levels were significantly higher in the model group than in the control group (*p* < 0.05; Figure 5A,B). Both adipokines were significantly lower in the LCP group than in the model group (*p* < 0.05).

The expression levels of the adipogenesis-related genes *Pparg* and *Cebpa* in WAT were significantly higher in the model group than in the control group (*p* < 0.05; Figure 5C,D). Compared with the model group, the LCP group showed significantly lower expression of both genes (*p* < 0.05). Overall, compared with the model group, the LCP group showed a more favorable serum lipid profile, lower circulating leptin and adiponectin levels, and lower WAT expression of *Pparg* and *Cebpa*, whereas body weight and WAT weight did not differ significantly between the two groups.

### 2.6. Exploratory Observations of Osteogenic and Adipogenic Differentiation in Group-Pooled BMSC Cultures

BMSCs established from bone marrow cells pooled from four mice within each experimental group were cultured under osteogenic or adipogenic induction conditions. Because each group was represented by a single pooled biological cell population, the results were summarized descriptively, without inferential statistical comparisons among groups.

**Figure 6:**
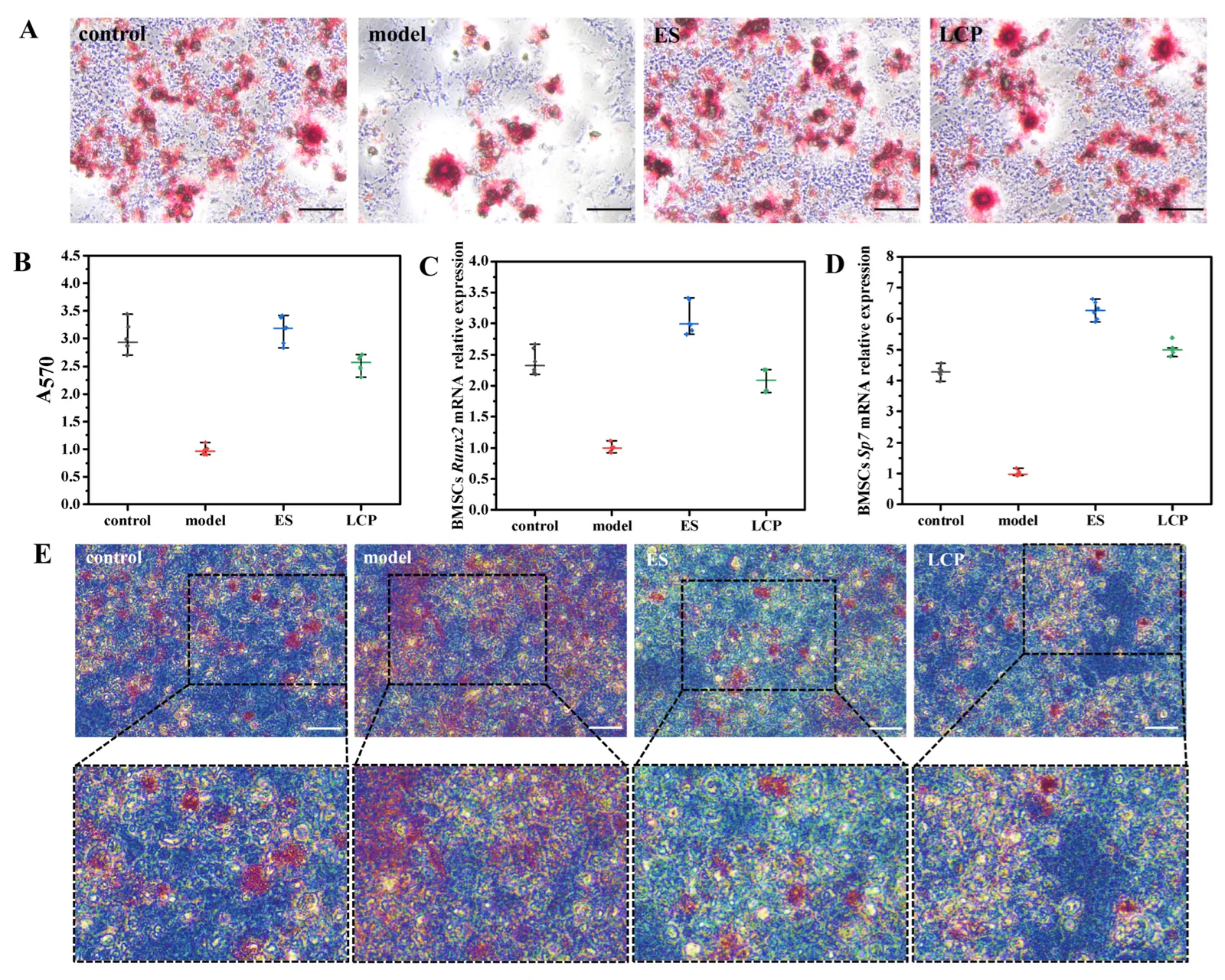

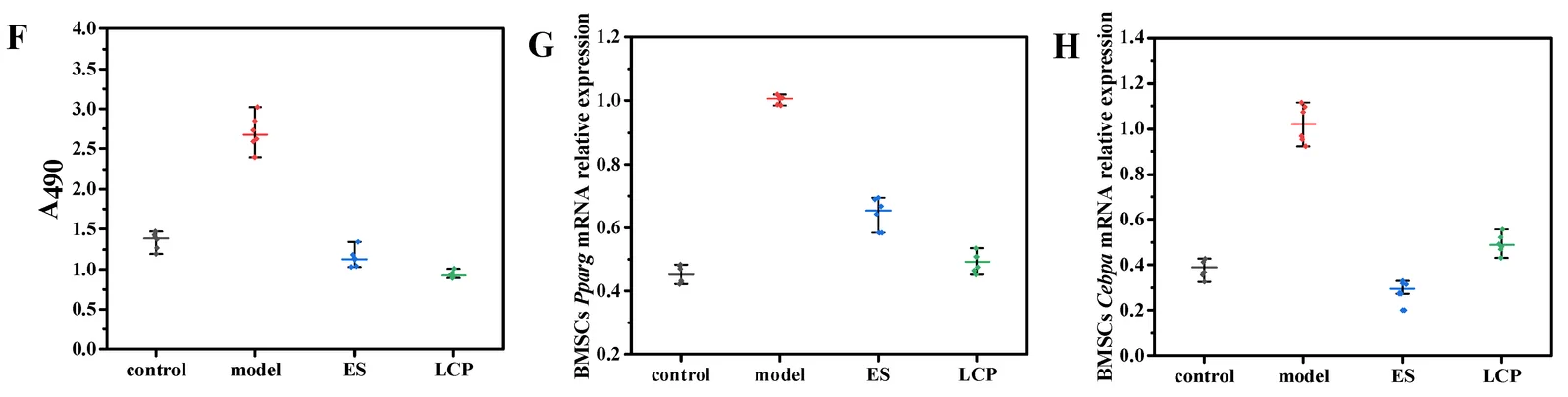
Exploratory observations of osteogenic and adipogenic differentiation in group-pooled BMSC cultures: (**A**) Alizarin Red S staining of mineralized nodules. (**B**) Quantitative analysis of mineralization by measurement of Alizarin Red S absorbance at 570 nm (A_570_). (**C**,**D**) RT-qPCR analysis of the osteogenesis-related genes *Runx2* (**C**) and *Sp7* (**D**). (**E**) Representative images of Oil Red O staining, with Oil Red O-positive lipid droplets appearing red. The (**lower panels**) show digitally magnified views of the boxed regions in the corresponding (**upper panels**) to facilitate visualization of lipid droplet accumulation. (**F**) Quantitative analysis of lipid accumulation by measurement of Oil Red O absorbance at 490 nm (A_490_). (**G**,**H**) RT-qPCR analysis of the adipogenesis-related genes *Pparg* (**G**) and *Cebpa* (**H**). Bone marrow cells from four mice within each experimental group were pooled before establishment of the BMSC cultures; consequently, each group was represented by a single pooled biological cell population. Quantitative data are presented descriptively. Each plotted point represents the mean of technical triplicate measurements from one of three repeated culture assays conducted using the same pooled BMSC population for that group, and the summary lines and error bars indicate the mean ± SD of the three assay-level values. These repeated culture assays and technical replicates were used to assess assay repeatability and were not considered independent biological replicates. No inferential statistical comparisons among groups were performed. Scale bars: 100 μm.

In the osteogenic assays, the pooled BMSC culture derived from mice in the model group showed less Alizarin Red S staining and a lower mean A_570_ value than the culture derived from mice in the control group. The cultures derived from mice in the ES and LCP groups showed greater staining and higher mean A_570_ values than the model-derived culture (Figure 6A,B). Mean *Runx2* and *Sp7* expression levels showed a similar pattern, with lower values in the model-derived culture and higher values in the ES- and LCP-derived cultures (Figure 6C,D).

In the adipogenic assays, the model-derived culture showed more Oil Red O staining and a higher mean A_490_ value than the control-derived culture. In comparison, the ES- and LCP-derived cultures showed less staining and lower mean A_490_ values than the model-derived culture (Figure 6E,F). Mean *Pparg* and *Cebpa* expression levels showed a corresponding pattern, with higher values in the model-derived culture and lower values in the ES- and LCP-derived cultures (Figure 6G,H).

Overall, compared with the model-derived pooled culture, the LCP-derived pooled culture showed greater Alizarin Red S staining and higher mean expression levels of the osteogenesis-related genes, together with less Oil Red O staining and lower mean expression levels of the adipogenesis-related genes. These observations are exploratory and require confirmation using independently derived biological replicates.

### 2.7. Associations Among Bone- and Lipid Metabolism-Related Parameters

**Figure 7:**
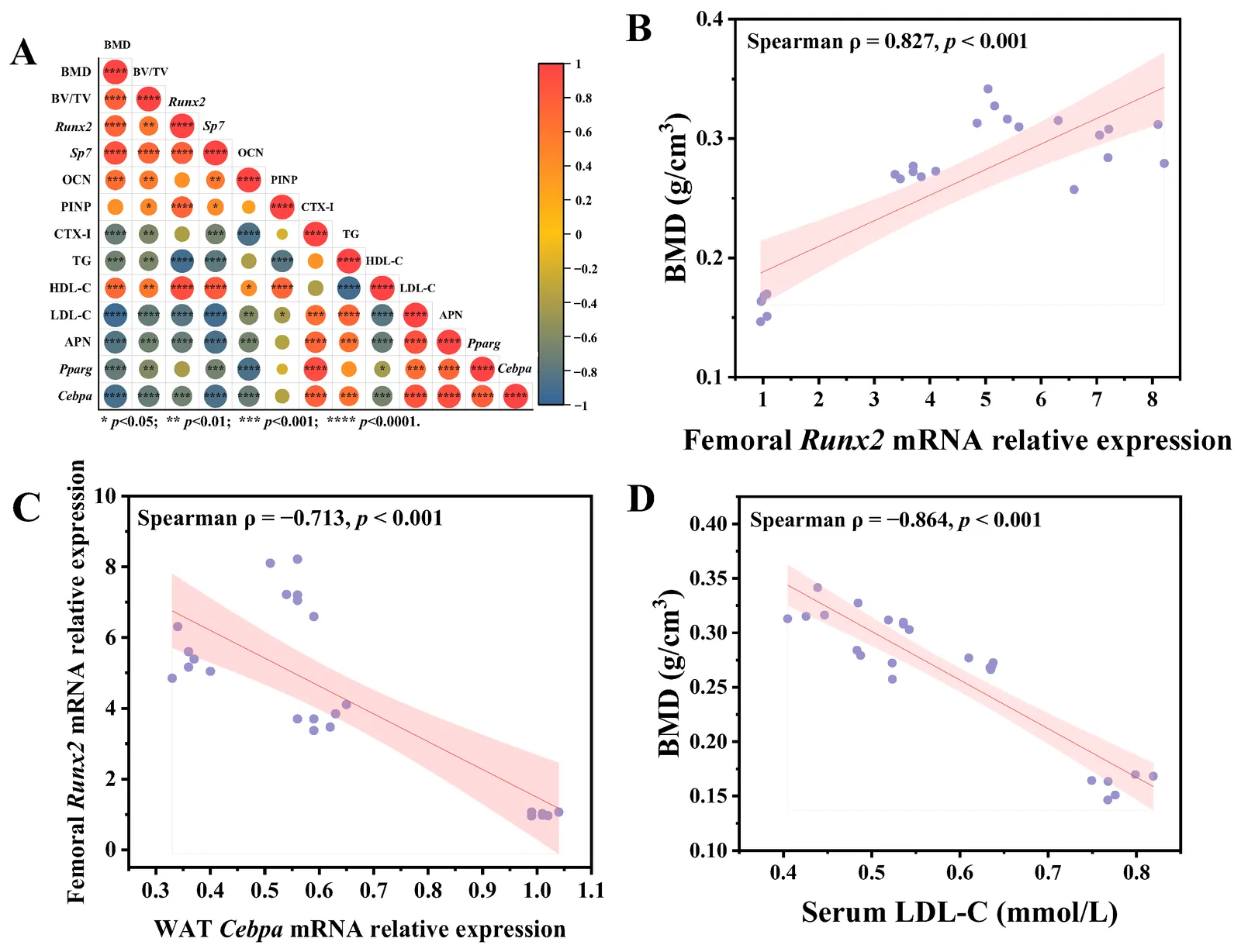
Correlation analysis among bone mass, osteogenesis-related, adipogenesis-related, and lipid metabolism-related indicators: (**A**) Heatmap of Spearman correlations among bone microstructure parameters, bone turnover markers, osteogenesis-related genes, adipogenesis-related genes, and lipid metabolism-related indicators. Circle color indicates the direction and magnitude of the correlations, whereas circle size reflects correlation strength. (**B**–**D**) Selected scatter plots showing correlations between BMD and femoral *Runx2* mRNA expression (**B**), femoral *Runx2* and WAT *Cebpa* mRNA expression (**C**), and BMD and serum LDL-C levels (**D**). Solid lines and shaded areas show linear regression fits and 95% confidence intervals for visualization; correlation coefficients and *p* values were obtained from Spearman correlation analysis. Asterisks indicate statistical significance: * *p* < 0.05, ** *p* < 0.01, *** *p* < 0.001, and **** *p* < 0.0001.

Spearman correlation analysis was performed among representative bone- and lipid metabolism-related parameters (Figure 7). The analyzed variables included the bone microarchitecture parameters BMD and BV/TV; femoral expression of the osteogenesis-related genes *Runx2* and *Sp7*; serum OCN, PINP, and CTX-I levels; serum TG, HDL-C, LDL-C, and adiponectin levels; and WAT expression of the adipogenesis-related genes *Pparg* and *Cebpa*.

The correlation heatmap showed that BMD and BV/TV were positively correlated with several osteogenesis-related indicators, including femoral *Runx2* and *Sp7* expression and serum OCN and PINP levels, and negatively correlated with several lipid metabolism- and adipogenesis-related indicators, including serum TG and LDL-C levels and WAT *Pparg* and *Cebpa* expression (Figure 7A). Negative correlations were also observed between several adipogenesis-related and osteogenesis-related indicators.

Selected scatter plots were generated to illustrate specific associations. BMD was positively correlated with femoral *Runx2* mRNA expression (Spearman’s ρ = 0.827, *p* < 0.001; Figure 7B). Femoral *Runx2* expression was negatively correlated with WAT *Cebpa* mRNA expression (Spearman’s ρ = −0.713, *p* < 0.001; Figure 7C). In addition, BMD was negatively correlated with serum LDL-C levels (Spearman’s ρ = −0.864, *p* < 0.001; Figure 7D). Collectively, these analyses identified associations among bone mass, osteogenesis-related, adipogenesis-related, and circulating lipid metabolism-related indicators.

## 3. Discussion

### 3.1. Attenuation of Trabecular Bone Deterioration by LCP Under Estrogen-Deficient Conditions

The marked reduction in serum E2 in the model group confirmed that repeated leuprorelin administration successfully induced an estrogen-deficient state, consistent with the pharmacological effects of sustained GnRH agonist administration reported previously [3,12]. Estradiol administration partially restored circulating E2 levels, whereas LCP administration did not significantly alter serum E2 levels relative to the model group. Thus, the preservation of trabecular bone microarchitecture observed with LCP occurred without restoration of circulating E2, indicating that the observed skeletal effects were not accompanied by an increase in circulating estrogen.

The micro-CT parameters evaluated in the present study provide complementary information on trabecular bone integrity. BMD reflects the mineralized density of bone, whereas BV/TV represents the proportion of trabecular bone within the analyzed volume and therefore reflects trabecular bone mass. Tb.N and Tb.Th characterize trabecular abundance and thickness, respectively, with decreases in these parameters indicating loss and thinning of trabeculae. Tb.Sp reflects the spacing between adjacent trabeculae, and an increase indicates expansion of intertrabecular spaces [13,14]. SMI characterizes trabecular morphology, with higher values generally reflecting a shift from a more plate-like to a more rod-like structure, a feature commonly associated with deterioration of trabecular architecture [15]. Accordingly, the reductions in BMD, BV/TV, Tb.N, and Tb.Th, together with the increases in Tb.Sp and SMI in the model group, indicate reduced trabecular bone mass, loss and thinning of trabeculae, increased trabecular separation, and deterioration of trabecular structural organization, rather than an isolated reduction in bone mineral density.

Concurrent LCP administration attenuated these microarchitectural alterations. BMD and BV/TV in the LCP group were comparable to those in the control group, while Tb.N and Tb.Th were higher and Tb.Sp and SMI were lower than those in the model group. Together, these changes indicate relative preservation of trabecular bone mass and microarchitecture. However, Tb.N and Tb.Th remained lower and SMI remained higher in the LCP group than in the control and ES groups, indicating partial rather than complete preservation of trabecular architecture. Consistent with the micro-CT findings, histological analysis of femoral metaphyseal sections showed a higher Tb.Ar in the LCP group than in the model group, providing complementary histological evidence of trabecular preservation. Because LCP administration began concurrently with leuprorelin treatment, these findings primarily reflect attenuation of developing trabecular deterioration rather than reversal of established bone loss.

These findings are broadly consistent with previous studies showing that food-derived calcium-binding or phosphorylated peptides can exert favorable effects on calcium utilization and bone-related outcomes [8,9]. Herring egg phosphopeptides have been reported to enhance calcium absorption and improve bone microarchitecture in vivo [10], whereas phosphorylated Antarctic krill peptides attenuated bone loss in a glucocorticoid-induced osteoporosis model [11]. The present findings extend these observations to a leuprorelin-induced estrogen-deficient model, in which concurrent LCP administration attenuated trabecular microarchitectural deterioration. The mechanisms underlying these effects remain to be clarified.

### 3.2. Attenuation of Bone Remodeling-Related Alterations by LCP Under Estrogen-Deficient Conditions

Bone remodeling depends on the balance between osteoblast-mediated bone formation and osteoclast-mediated bone resorption [16]. Serum bone turnover markers provide complementary biochemical information regarding these processes [16,17]. PINP reflects type I collagen synthesis during bone matrix formation, BALP is associated with osteoblast differentiation and matrix mineralization, and OCN is a non-collagenous protein produced predominantly by osteoblasts and commonly used as an indicator of bone formation-related activity. In contrast, CTX-I is released during type I collagen degradation and is widely used as a systemic marker of bone resorption [16,17]. Therefore, the concomitant reductions in PINP, OCN, and BALP and the elevation of CTX-I in the model group were consistent with an unfavorable bone turnover profile characterized by reduced bone formation-related activity and increased bone resorption-related collagen degradation. These biochemical alterations were also consistent with the deterioration of trabecular microarchitecture observed by micro-CT.

Compared with the model group, the LCP group showed higher serum PINP, OCN, and BALP levels and a lower CTX-I level. This interpretation was further supported by the bone tissue gene expression and immunohistochemical findings. *Runx2* and *Sp7* are key transcriptional regulators of osteoblast lineage commitment and differentiation [18,19]. Their reduced expression in femoral tissue from the model group was consistent with a less favorable osteogenesis-related transcriptional profile, whereas their higher expression in the LCP group than in the model group was consistent with a more favorable osteogenesis-related transcriptional profile. Similarly, the increased OCN immunoreactivity in tibial tissue from the LCP group was consistent with the higher circulating OCN level. Together, these biochemical, transcriptional, and tissue-level findings indicate that the preservation of trabecular microarchitecture observed with LCP was accompanied by a more favorable bone formation-related profile.

The osteoclast-associated transcriptional response was selective. *Tnfsf11* encodes RANKL, an essential regulator of osteoclastogenesis that is produced by several skeletal mesenchymal cell populations, including osteogenic and marrow adipogenic/stromal lineage cells, rather than representing an osteoclast-specific marker [20,21]. *Nfkb1* encodes a component of the NF-κB signaling pathway involved in RANKL-induced osteoclastogenic regulation; however, NF-κB signaling is not restricted to osteoclasts, and *Nfkb1* expression in whole bone therefore cannot be considered an osteoclast-specific readout [22]. No significant differences in the expression of either gene were observed among the groups. In contrast, *Nfatc1*, a central transcriptional regulator of osteoclast differentiation, and *Ctsk*, which encodes a protease involved in bone matrix degradation, were increased in the model group relative to the control group and were lower in the LCP group than in the model group [23]. However, *Dcstamp*, which is associated with osteoclast precursor fusion, and *Acp5*, which encodes tartrate-resistant acid phosphatase, did not differ significantly among the groups [23,24]. These divergent responses indicate selective rather than uniform changes in osteoclast-associated transcription. Together with the lower serum CTX-I level, the reductions in *Nfatc1* and *Ctsk* are consistent with attenuation of selected osteoclast-associated and bone resorption-related alterations. Nevertheless, because CTX-I is a systemic marker and osteoclast number and function were not directly assessed, the present data do not establish direct inhibition of osteoclast formation or resorptive activity.

Serum calcium and phosphorus levels did not differ significantly among the groups, indicating that neither leuprorelin administration nor concurrent LCP administration significantly altered circulating calcium or phosphorus concentrations under the present experimental conditions. Because circulating calcium and phosphorus are tightly regulated, these unchanged levels do not exclude possible effects on calcium absorption, utilization, or local skeletal mineralization [25,26]. Overall, the preservation of trabecular microarchitecture observed with LCP was accompanied by a more favorable bone formation-related profile and attenuation of selected osteoclast-associated and bone resorption-related alterations.

### 3.3. Parallel Changes in Marrow Adiposity and Systemic Lipid Metabolism, with Exploratory Observations from Group-Pooled BMSC Cultures

Bone marrow adipose tissue is closely associated with the skeletal microenvironment, and increased marrow adiposity frequently accompanies impaired bone formation and trabecular bone loss under estrogen-deficient conditions [27,28]. In the present study, the model group exhibited increased marrow Ad.Ar together with higher femoral expression of the adipogenesis-related genes *Pparg* and *Cebpa*, whereas both marrow Ad.Ar and the expression of these genes were lower in the LCP group. Together, these findings indicate a less adipogenic bone marrow profile in the LCP group, occurring in parallel with preservation of trabecular microarchitecture.

Systemic metabolic changes showed a similar overall pattern. Body weight remained comparable among the groups throughout the experimental period, whereas WAT weight was higher in the model group than in the control and ES groups. Although mean WAT weight was lower in the LCP group than in the model group, this difference was not statistically significant, indicating that the lipid-related changes observed in the LCP group occurred without a significant reduction in overall WAT mass. Nevertheless, the LCP group exhibited lower serum TC, TG, and LDL-C levels and higher HDL-C levels than the model group, together with lower circulating leptin and adiponectin levels. Because leptin and adiponectin exert distinct and context-dependent effects on energy metabolism, adipose endocrine function, and bone metabolism [29,30,31], their concurrent elevation in the model group is best described as an altered adipokine profile rather than a uniform directional change in adipokine activity. In parallel, WAT expression of *Pparg* and *Cebpa* was lower in the LCP group than in the model group, consistent with lower adipogenesis-related gene expression despite the absence of a significant reduction in WAT weight.

Exploratory observations from the group-pooled BMSC cultures were directionally consistent with these in vivo findings. The pooled culture derived from model-group mice showed lower mineralization and osteogenesis-related gene expression, together with greater lipid accumulation and adipogenesis-related gene expression, whereas the pooled culture derived from LCP-administered mice showed the opposite descriptive pattern. These observations paralleled the higher femoral expression of *Runx2* and *Sp7*, lower expression of *Pparg* and *Cebpa*, and lower marrow Ad.Ar observed in the LCP group. Previous studies have similarly reported altered osteogenic and adipogenic differentiation properties in BMSCs isolated from estrogen-deficient animals during subsequent ex vivo culture [32,33]. However, because bone marrow cells were pooled within each group before culture, each group was represented by only one biological cell population. The repeated culture assays and technical triplicates did not constitute independent biological replication. Therefore, the present BMSC findings should be interpreted only as exploratory, hypothesis-generating observations and cannot establish treatment effects at the animal level. Confirmation using independently established BMSC cultures from individual animals is required.

The calcium-chelating property of LCP is a physicochemical characteristic of the peptide preparation, whereas the mechanisms underlying the lipid metabolism- and adipogenesis-related changes observed in this study have not been established. Following oral administration, food-derived peptide components may undergo gastrointestinal digestion and metabolic transformation, and intact peptides or peptide-derived products may be absorbed to varying extents [9,34,35]. Therefore, the calcium-chelating capacity of LCP alone cannot be assumed to account for the systemic lipid metabolism- and adipogenesis-related changes observed in this study. Whether these changes reflect direct actions of peptide-derived components or indirect effects arising from alterations in the systemic or bone marrow environment remains unclear.

Collectively, attenuation of trabecular bone deterioration by LCP occurred in parallel with lower marrow adiposity, a more favorable circulating lipid profile, altered adipokine levels, and lower adipogenesis-related gene expression in femoral tissue and WAT. Exploratory observations from group-pooled BMSC cultures showed directionally consistent differentiation-related patterns, but these findings require confirmation using independent biological replicates.

### 3.4. Associations Between Skeletal and Lipid Metabolism-Related Outcomes

Bone and lipid metabolism are biologically interconnected through multiple systemic, endocrine, and cellular processes [36,37]. In the present study, the correlation heatmap showed that higher BMD and BV/TV were generally associated with more favorable osteogenesis-related profiles, including higher femoral *Runx2* and *Sp7* expression and higher serum OCN and PINP levels. Conversely, BMD and BV/TV were negatively associated with several lipid metabolism- and adipogenesis-related indicators, including serum TG and LDL-C levels and WAT *Pparg* and *Cebpa* expression. Overall, lower BMD and BV/TV were associated with lower osteogenesis-related indicators and higher levels of several adipogenesis- and lipid metabolism-related indicators.

The selected correlations further illustrated these relationships. The positive association between BMD and femoral *Runx2* expression was consistent with the established role of *Runx2* in osteoblast differentiation and bone formation [22,23]. The negative correlation between femoral *Runx2* and WAT *Cebpa* expression further illustrated the inverse association between osteogenesis- and adipogenesis-related indicators across bone and adipose tissue. Similarly, the negative association between BMD and serum LDL-C was consistent with observational studies reporting inverse associations between LDL-C and BMD in certain populations [38,39]. Collectively, these correlations provide an integrated view of the parallel skeletal and metabolic changes observed in the present study, although their directionality and mechanistic relationships remain unresolved.

### 3.5. Limitations and Future Directions

The present findings support further investigation of LCP as a food-derived nutritional ingredient for supporting bone health under estrogen-deficient conditions. Because LCP administration was initiated concurrently with model induction, the present study evaluated attenuation of developing bone loss rather than treatment of established osteoporosis. Accordingly, the findings are most relevant to the potential complementary nutritional use of LCP under estrogen-deficient conditions.

Several limitations should be considered. The study was conducted exclusively in young female mice with pharmacologically induced estrogen deficiency; therefore, whether LCP would exert similar effects in naturally occurring age-related osteoporosis, particularly in males, remains unknown [40,41]. Only one LCP dose and one intervention duration were examined, and LCP consisted of a complex peptide hydrolysate rather than defined purified peptides. The active peptide sequences, gastrointestinal stability, bioavailability, calcium absorption and utilization, and long-term safety were not determined. Because bone marrow cells were pooled within each experimental group before culture, the BMSC experiments included only one biological cell population per group. The repeated culture assays and technical triplicates were not independent biological replicates; therefore, no inferential statistical conclusions can be drawn from the BMSC data, which should be interpreted solely as exploratory and descriptive observations. In addition, osteoclast number and resorptive activity were not directly assessed, and the present biochemical and transcriptional data therefore provide only indirect evidence regarding bone resorption-related changes. More broadly, the current histological, biochemical, and transcriptional findings, together with the exploratory BMSC observations, do not establish the direct cellular or molecular mechanisms underlying the skeletal and metabolic responses to LCP.

Future studies should identify the active peptide components of LCP, characterize their gastrointestinal stability and bioavailability, establish dose–response relationships and long-term safety, and directly evaluate their effects in relevant bone and adipose cell models. In particular, the BMSC observations should be validated using independently established BMSC cultures from individual animals. Further validation using therapeutic intervention designs, naturally aged female and male animals, and additional osteoporosis models will also be important for determining whether the present findings extend beyond pharmacologically induced estrogen deficiency and for evaluating the translational potential of LCP as a food-derived nutritional ingredient for supporting bone health.

## 4. Materials and Methods

### 4.1. Materials

*Larimichthys crocea* was purchased from Yonghui Supermarket in Ningde (Ningde, China). Leuprorelin acetate microspheres for injection were obtained from Beijing Biote Pharmaceutical Co., Ltd. (Beijing, China). Estradiol tablets were purchased from Zhejiang Xianju Pharmaceutical Co., Ltd. (Taizhou, China). Commercial ELISA kits for estradiol (E2), type I collagen cross-linked C-terminal telopeptide (CTX-I), osteocalcin (OCN), adiponectin (APN), leptin, and procollagen type I N-terminal propeptide (PINP) were purchased from Cloud-Clone Corp. (Wuhan, China). Commercial assay kits for bone alkaline phosphatase (BALP), calcium (Ca), phosphorus (P), total cholesterol (TC), triglyceride (TG), high-density lipoprotein cholesterol (HDL-C), and low-density lipoprotein cholesterol (LDL-C) were purchased from Nanjing Jiancheng Bioengineering Institute (Nanjing, China). Catalogue numbers and manufacturer-reported analytical performance characteristics of these assay kits are provided in Supplementary Table S2. Oil Red O staining kit was purchased from Beyotime Biotechnology Co., Ltd. (Shanghai, China). Dexamethasone (DEX), β-glycerophosphate (BGP), Alizarin Red S, insulin, and isopropanol were purchased from Shanghai Yuanye Biological Technology Co., Ltd. (Shanghai, China). All the other chemicals and reagents were of analytical grade and commercially available.

### 4.2. Preparation of Larimichthys crocea-Derived Calcium-Chelating Peptides

Surimi prepared from eviscerated *Larimichthys crocea* was used as the raw material. Briefly, 10 g of surimi was mixed with ultrapure water to obtain a substrate concentration of 7% (*w*/*v*). The pH of the mixture was adjusted to 6.0, and neutral protease (100 U/mg, Cat. No. S10013; Shanghai Yuanye Biological Technology Co., Ltd., Shanghai, China) was added at an enzyme-to-substrate ratio of 6% (*w*/*w*). Enzymatic hydrolysis was performed in a water bath shaker at 40 °C for 2 h. The reaction mixture was then heated in a boiling water bath for 10 min to inactivate the enzyme, followed by centrifugation at 10,000× *g* for 20 min at 4 °C. The supernatant was collected and lyophilized to obtain *Larimichthys crocea* protein hydrolysates. The calcium-chelating capacity of the lyophilized hydrolysates was determined using the o-cresolphthalein complexone colorimetric method as previously described [42] and was found to be 69.10 μg/mg. The resulting peptide hydrolysate with calcium-chelating capacity was designated as *Larimichthys crocea*-derived calcium-chelating peptides (LCP) and used in the subsequent animal experiments. No exogenous calcium was added to the LCP preparation used for animal administration.

### 4.3. Animal Experiment Design

Female ICR mice (5 weeks old, 18–22 g) were purchased from Shanghai SLAC Laboratory Animal Co., Ltd. (Shanghai, China) and housed in the Animal Experiment Center of Ningde Normal University. The mice were maintained under specific pathogen-free (SPF) conditions at 24 ± 1 °C and 55 ± 5% relative humidity with a 12 h light/dark cycle. Standard chow and water were provided *ad libitum*. All the animal experiments were performed in accordance with the Guidelines for the Care and Use of Laboratory Animals and were approved by the Animal Care and Use Committee of Ningde Normal University (Approval No. NDNU-LL-202507).

After one week of acclimatization, the mice were randomly divided into four groups, with 10 mice in each group: control group (control), model group (model), estradiol positive-control group (ES), and LCP group (LCP). The overall experimental design is shown in Supplementary Figure S1. The leuprorelin-induced estrogen-deficiency protocol was established with reference to a previously reported GnRH agonist-induced bone-loss model, with modifications to the experimental duration and administration procedure [3,12]. Mice in the model, ES, and LCP groups were intramuscularly injected with leuprorelin acetate microspheres at a dose of 375 μg per mouse once every 4 weeks for a total of three injections, whereas mice in the control group received an equivalent volume of normal saline by intramuscular injection. Beginning on the same day as the first leuprorelin administration, mice in the ES group received estradiol solution at a dose of 50 μg/kg body weight, and mice in the LCP group received LCP solution at a dose of 200 mg/kg body weight. Mice in the control and model groups received an equivalent volume of normal saline by oral gavage. All the oral administrations were performed once daily for 12 weeks, with a gavage volume of approximately 0.2 mL per mouse. Body weight was recorded daily, and the body-weight-based gavage doses were adjusted accordingly. For presentation and statistical comparison of body-weight changes over time, measurements at weeks 0, 4, 8, and 12 were used. Thus, ES and LCP administration was initiated concurrently with leuprorelin-induced model induction, representing a concurrent intervention design rather than treatment after establishment of bone loss.

At the end of the 12-week experimental period, mice from each group were allocated to different analyses. Six mice per group were used for serum biochemical assays, micro-computed tomography (micro-CT) analysis, histological and immunohistochemical analyses, and tissue gene expression analysis. Blood samples were collected under anesthesia, and serum was separated for biochemical measurements. For skeletal analyses, the left tibia was collected for micro-CT analysis, the right tibia for OCN immunohistochemistry, the left femur for H&E staining, and the right femur for gene expression analysis. Inguinal white adipose tissue (WAT) was also collected and weighed before subsequent processing. Samples were fixed or stored at −80 °C according to the requirements of the respective analyses. Bone marrow cells collected from the remaining four mice within each group were pooled for the isolation and culture of bone marrow mesenchymal stem cells (BMSCs).

### 4.4. Micro-CT Scanning

Tibial trabecular microarchitecture was assessed using a high-resolution micro-CT system (SkyScan 1276, Bruker microCT, Kontich, Belgium). The left tibias were scanned at an X-ray source voltage of 80 kV and a current of 124 μA, with a resolution of 8 μm. Projection images were reconstructed using NRecon software (version 1.7.4.2), and the reconstructed datasets were reoriented using DataViewer software (version 1.5.6.2). Trabecular bone parameters in the proximal tibia were analyzed using CTAn software (version 1.20.8.0), while three-dimensional images were generated using CTVox software (version 3.3.0; all software from Bruker microCT, Kontich, Belgium). The analyzed parameters included bone mineral density (BMD, g/cm^3^), bone volume fraction (BV/TV, %), structure model index (SMI), trabecular number (Tb.N, 1/mm), trabecular separation (Tb.Sp, mm), and trabecular thickness (Tb.Th, mm).

### 4.5. Determination of Serum Biochemical Markers

Serum E2, PINP, OCN, CTX-I, leptin, and adiponectin levels were measured using commercial ELISA kits (Cloud-Clone Corp., Wuhan, China) according to the manufacturers’ instructions. Serum BALP, Ca, P, TC, TG, HDL-C, and LDL-C levels were determined using the corresponding commercial assay kits (Nanjing Jiancheng Bioengineering Institute, Nanjing, China) according to the manufacturers’ protocols. The manufacturers, catalogue numbers, assay methods, detection or linear ranges, sensitivity or limit-of-detection (LOD) information, and precision characteristics, where available in the manufacturer documentation, are summarized in Supplementary Table S2.

### 4.6. H&E Staining

The left femurs were fixed in 4% paraformaldehyde for 72 h and decalcified in 15% EDTA decalcifying solution (pH 7.2; Solarbio, Beijing, China) at room temperature for 2 weeks, with the decalcifying solution replaced once per week. After decalcification, the samples were dehydrated through a graded ethanol series, cleared in xylene, embedded in paraffin, and cut into 4–5 μm thick sections. For H&E staining, the sections were deparaffinized in xylene, rehydrated through a descending ethanol series, stained with hematoxylin for 5 min, rinsed with deionized water, and stained with eosin for 2 min. After staining, the sections were dehydrated, cleared, and mounted with neutral resin. Microscopic fields were selected from anatomically comparable regions of the metaphysis adjacent to the growth plate across all groups. Within these predefined regions, three microscopic fields per animal were randomly selected for quantitative histological analysis. Trabecular bone area (Tb.Ar) and marrow adipocyte area (Ad.Ar) were quantified using ImageJ software (version 1.54; National Institutes of Health, Bethesda, MD, USA). The mean value obtained from the three fields was used as the representative value for each animal.

### 4.7. Immunohistochemical Analysis

The right tibias were fixed in 4% paraformaldehyde for 48 h and decalcified in 15% EDTA decalcifying solution (pH 7.2; Solarbio, Beijing, China) at room temperature for 2 weeks, with the decalcifying solution replaced once per week. After decalcification, the samples were dehydrated through a graded ethanol series, cleared in xylene, embedded in paraffin, and cut into 4–5 μm thick sections. The sections were deparaffinized in xylene and rehydrated through a descending ethanol series. Antigen retrieval was performed using citrate antigen retrieval solution (Solarbio, Beijing, China) at 95 °C for 10 min. Sections were then incubated overnight at 4 °C with a rabbit polyclonal antibody against osteocalcin (OCN; Invitrogen, Thermo Fisher Scientific, Waltham, MA, USA; Cat. No. PA5-96529; 1:200). The sections were subsequently incubated for 30 min at room temperature with an alkaline phosphatase (AP)-polymer anti-rabbit IgG detection reagent and developed using the Permanent Red chromogen/substrate system, both supplied with the DoubleStain IHC Kit (Cat. No. ab210061; Abcam, Cambridge, UK). Following hematoxylin counterstaining, the sections were mounted and examined under a light microscope. Microscopic fields were selected from anatomically comparable regions of the metaphysis adjacent to the growth plate across all groups. Within these predefined regions, three microscopic fields per animal were randomly selected for quantitative analysis. The integrated optical density (IOD) of OCN-positive staining was quantified using ImageJ software. The mean IOD value from the three fields was used as the representative value for each animal.

### 4.8. Isolation and Culture of BMSCs

BMSCs were isolated and cultured as previously described [1] with modifications. At the end of the 12-week experimental period, the bilateral femurs and tibias of four mice from each group were collected under sterile conditions, and surrounding muscles and connective tissues were carefully removed. The bones were placed in 6-well plates containing ice-cold PBS. Both ends of each bone were cut to expose the marrow cavity. Bone marrow cells were separately flushed from the femurs and tibias of each mouse with complete culture medium (DMEM supplemented with 10% FBS and 1% penicillin/streptomycin) using a sterile 1-mL syringe fitted with a 0.45 × 16 mm needle (KDL; Cat. No. 60017031; Shanghai Kindly Enterprise Development Group Co., Ltd., Shanghai, China). For each group, the resulting cell suspensions from the four mice were then combined to form a single pooled bone marrow cell population, which was cultured at 37 °C in a humidified atmosphere containing 5% CO_2_. After 24 h, the medium was replaced to remove non-adherent cells, and adherent cells were cultured with medium renewal every 2 days. When the cells reached approximately 90–95% confluence, they were passaged. Third-passage BMSCs (P3) derived from the single pooled bone marrow cell population for each group were used for the subsequent exploratory BMSC assays. Three repeated culture assays were conducted using each group-pooled BMSC population, with each assay performed in technical triplicate. These repeated culture assays and technical triplicates were used only to assess assay repeatability and were not considered independent biological replicates.

### 4.9. Osteogenic Differentiation

Osteogenic differentiation was induced as previously described [1], with modifications. Third-passage BMSCs (P3) were plated into 6-well plates at a density of 1 × 10^5^ cells/well and cultured at 37 °C in a humidified incubator with 5% CO_2_ until cell attachment. When the cell monolayer reached approximately 80% confluence, the basal medium was replaced with osteogenic induction medium composed of DMEM supplemented with 10% FBS, 1% penicillin/streptomycin, 5 mM BGP, 100 nM DEX, and 50 μM ascorbic acid. The induction culture was continued for 21 days, with medium renewal every other day. At the end of the induction period, cells were washed and fixed with 4% paraformaldehyde for 10 min at room temperature. Calcium deposition was evaluated by staining the cells with 40 mM Alizarin Red S solution (pH 4.2) for 30 min. After staining, excess dye was removed by rinsing several times with deionized water, and representative images were captured using a light microscope. For quantitative analysis, the mineral-bound dye was extracted with 10% cetylpyridinium chloride for 1 h at room temperature in the dark, and the absorbance of the extracted solution was measured at 570 nm using a microplate reader. Each assay was performed in technical triplicate.

### 4.10. Adipogenic Differentiation

Adipogenic differentiation was induced as previously described [1], with modifications. Third-passage BMSCs (P3) were seeded in 6-well plates at a density of 1 × 10^5^ cells/well and cultured at 37 °C in a humidified atmosphere containing 5% CO_2_. When the cells reached approximately 90% confluence, the culture medium was replaced with fresh complete medium, and the cells were maintained for an additional 2 days. Adipogenic induction was then initiated using complete adipogenic induction medium (DMEM supplemented with 10% FBS, 10 μg/mL insulin, 5 μM troglitazone, 1 μM DEX, 0.5 mM IBMX, and 1% penicillin/streptomycin) for 2 days. The medium was subsequently replaced with adipogenic maintenance medium (DMEM supplemented with 10% FBS, 10 μg/mL insulin, and 1% penicillin/streptomycin) for 8 days, with medium renewal every other day. At the end of the induction period, cells were fixed with 4% paraformaldehyde for 20 min at room temperature and washed with 60% isopropanol for 30 s. Lipid droplets were visualized by staining with filtered Oil Red O solution according to the manufacturer’s instructions, and representative images were captured under a light microscope. For quantification, the stained dye was extracted with isopropanol, and the absorbance of the extracted solution was measured at 490 nm using a microplate reader. Each assay was conducted in technical triplicate.

### 4.11. Correlation Analysis

Correlation analysis was performed to explore associations among bone parameters, serum bone turnover markers, lipid metabolism-related markers, and gene expression levels. The analyzed variables included BMD and BV/TV; serum bone turnover markers (OCN, PINP, and CTX-I); serum lipid metabolism-related indicators (TC, TG, HDL-C, LDL-C, leptin, and adiponectin); femoral osteogenesis-related gene expression (*Runx2* and *Sp7*); and WAT adipogenesis-related gene expression (*Pparg* and *Cebpa*). Spearman’s rank correlation analysis was used because the sample size was relatively limited and the variables were not assumed to follow a normal distribution.

Correlation coefficients were calculated among the selected indicators and visualized using a correlation heatmap, in which the color intensity represented the strength and direction of correlations. Statistical significance was indicated on the heatmap as * *p* < 0.05, ** *p* < 0.01, and *** *p* < 0.001. Representative scatter plots were generated to illustrate key associations related to bone formation and lipid metabolism, including correlations between BMD and femoral *Runx2* mRNA expression, femoral *Runx2* mRNA expression and WAT *Cebpa* mRNA expression, and BMD and serum LDL-C level. Linear regression lines with 95% confidence intervals were added to the scatter plots. All the correlation analyses and graphical visualizations were performed using OriginPro 2021, and a value of *p* < 0.05 was considered statistically significant.

### 4.12. RT-qPCR

RT-qPCR was performed to determine the mRNA expression levels of selected genes in femoral tissue, BMSCs, and WAT. For femoral tissue, the analyzed genes included the osteogenesis-related genes *Runx2* and *Sp7*, the osteoclastogenesis-related regulatory genes *Tnfsf11* and *Nfkb1*, the osteoclast-associated genes *Nfatc1*, *Ctsk*, *Dcstamp*, and *Acp5*, and the adipogenesis-related genes *Pparg* and *Cebpa*. For BMSCs, osteogenic and adipogenic differentiation-related genes, including *Runx2*, *Sp7*, *Pparg*, and *Cebpa*, were analyzed. For WAT, adipogenesis-related genes, including *Pparg* and *Cebpa*, were determined.

Briefly, total RNA was extracted from the collected samples using TRIzol reagent (Invitrogen, Carlsbad, CA, USA) according to the manufacturer’s instructions. RNA concentration and purity were assessed using a spectrophotometer based on the absorbance ratio at 260/280 nm. Subsequently, 1 μg of total RNA was reverse-transcribed into cDNA using a commercial reverse transcription kit (Takara Bio Inc., Shiga, Japan). RT-qPCR was performed using SYBR Green Master Mix (Applied Biosystems, Foster City, CA, USA) on a QuantStudio 6 Flex Real-Time PCR System (Applied Biosystems). The amplification program consisted of initial denaturation at 95 °C for 5 min, followed by 40 cycles of denaturation at 95 °C for 10 s and annealing/extension at 60 °C for 30 s. The relative mRNA expression levels of target genes were normalized to the reference gene *Gapdh* and calculated using the 2^−ΔΔCt^ method. For femoral and WAT samples, each mouse served as an independent biological replicate. For BMSC-related assays, RNA was extracted from P3 BMSCs obtained from bone marrow cells pooled from four mice within each group. All the qPCR reactions were performed in technical triplicate. Primer sequences for the target and reference genes are listed in Supplementary Table S3.

### 4.13. Statistical Analysis

Animal-level data are expressed as the mean ± standard deviation (SD). Statistical analyses were performed using SPSS 26.0 software (IBM Corp., Armonk, NY, USA). Differences among groups for animal-level measurements were assessed using one-way analysis of variance (ANOVA), followed by Duncan’s multiple range test for post hoc comparisons. A value of *p* < 0.05 was considered statistically significant. For the BMSC experiments, bone marrow cells from four mice within each group were pooled before culture, resulting in one pooled biological cell population per group. The repeated culture assays and technical triplicates were used only to assess assay repeatability and were not treated as independent biological replicates. The BMSC results were summarized descriptively, and no inferential statistical comparisons among groups were performed.

## 5. Conclusions

In this study, *Larimichthys crocea*-derived calcium-chelating peptides (LCP) attenuated leuprorelin-induced trabecular bone deterioration in estrogen-deficient mice. LCP administration was accompanied by more favorable bone formation-related profiles, attenuation of selected bone resorption-related alterations, reduced marrow adiposity, improved serum lipid profiles, and lower adipogenesis-related gene expression. Exploratory assays using group-pooled BMSC cultures showed differentiation-related patterns that were directionally consistent with these in vivo observations, but these descriptive findings require confirmation using independent biological replicates. Correlation analysis further revealed associations among bone mass, osteogenesis-related indicators, and lipid metabolism-related parameters. Together, these findings show that attenuation of estrogen deficiency-related bone loss by LCP occurred alongside parallel skeletal and metabolic changes, while the causal relationships among these responses remain to be established. These results support further investigation of LCP as a food-derived nutritional ingredient or complementary nutritional strategy for supporting bone health under estrogen-deficient conditions.

## Figures and Tables

**Table 1:** Serum lipid profiles in estrogen-deficient mice.

Group	TC/(mmol/L)	TG/(mmol/L)	LDL-C/(mmol/L)	HDL-C/(mmol/L)
Control	2.01 ± 0.10 ^c^	1.12 ± 0.05 ^b^	1.46 ± 0.05 ^b^	2.03 ± 0.07 ^ab^
Model	2.61 ± 0.08 ^a^	1.36 ± 0.02 ^a^	1.78 ± 0.03 ^a^	1.52 ± 0.04 ^c^
ES	1.97 ± 0.05 ^c^	0.89 ± 0.04 ^c^	1.51 ± 0.03 ^b^	2.18 ± 0.09 ^a^
LCP	2.25 ± 0.06 ^b^	1.18 ± 0.04 ^b^	1.61 ± 0.04 ^b^	1.84 ± 0.05 ^b^

Note: Data are presented as mean ± SD (*n* = 6). Different lowercase letters within the same column indicate significant differences among groups (*p* < 0.05).

## Data Availability

The raw data supporting the conclusions of this article will be made available by the authors upon request.

## Supplementary Materials

The following supporting information can be downloaded at: https://www.mdpi.com/article/10.3390/md24090301/s1, Figure S1: Schematic illustration of the experimental design in leuprorelin-induced estrogen-deficient mice; Figure S2: Serum estradiol (E2) levels in leuprorelin-induced estrogen-deficient mice; Figure S3: Effects of LCP on additional osteoclast-associated gene expression in femoral bone tissue; Table S1: Effects of LCP on body weight and WAT weight in estrogen-deficient mice; Table S2: Commercial assay kits used for serum biochemical measurements and their manufacturer-reported performance characteristics; Table S3: Primer sequences used for RT-qPCR.

## Abbreviations

The following abbreviations are used in this manuscript:

*Acp5*	Acid phosphatase 5, tartrate resistant
Ad.Ar	Marrow adipocyte area
APN	Adiponectin
BALP	Bone alkaline phosphatase
BMD	Bone mineral density
BMSCs	Bone marrow mesenchymal stem cells
BV/TV	Bone volume fraction
*C**ebpa*	CCAAT/enhancer-binding protein alpha
*Ctsk*	Cathepsin K
CTX-I	C-terminal telopeptide of type I collagen
*Dcstamp*	Dendrocyte-expressed seven-transmembrane protein
E2	Estradiol
ES	Estradiol positive-control group
HDL-C	High-density lipoprotein cholesterol
IOD	Integrated optical density
LDL-C	Low-density lipoprotein cholesterol
LCP	*Larimichthys crocea*-derived calcium-chelating peptides
micro-CT	Micro-computed tomography
*Nfatc1*	Nuclear factor of activated T cells 1
*Nfkb1*	Nuclear factor kappa B subunit 1
OCN	Osteocalcin
PINP	Procollagen type I N-terminal propeptide
*P**parg*	Peroxisome proliferator-activated receptor gamma
*Runx2*	Runt-related transcription factor 2
SMI	Structure model index
*Sp7*	*Sp7* transcription factor
Tb.Ar	Trabecular bone area
Tb.N	Trabecular number
Tb.Sp	Trabecular separation
Tb.Th	Trabecular thickness
TC	Total cholesterol
TG	Triglyceride
*Tnfsf11*	*Tnfsf11*
WAT	White adipose tissue
