# Supplementary material for: Larimichthys crocea-Derived Calcium-Chelating Peptides Attenuate Leuprorelin-Induced Bone Loss and Lipid Metabolic Alterations in Mice
Source: Mar Drugs. 2026 Aug 30;24(9):301. doi: 10.3390/md24090301 (PMC13608619; doi:10.3390/md24090301)
Supplement: Supplementary file 1 [file marinedrugs-24-00301-s001.zip › marinedrugs-4411809-supplementary.pdf]

Supporting information

Table S1 Effects of LCP on body weight and WAT weight in estrogen-deficient mice

Group	Body weight (g)				WAT weight (g)
	0 week	4th week	8th week	12th week	
control	20.67±0.89 a	26.46±1.12 a	29.32±1.23 a	29.76±1.91 a	0.25±0.02 b
model	21.18±1.24 a	29.25±1.87 a	33.44±1.17 a	33.39±2.10 a	0.37±0.02 a
ES	20.79±1.41 a	28.58±2.04 a	30.18±2.01 a	29.17±1.28 a	0.26±0.03 b
LCP	21.25±1.56 a	28.31±1.79 a	28.45±1.82 a	29.49±2.04 a	0.30±0.02 ab

Note: Data are presented as mean ± SD (n=6). Different lowercase letters within the same column indicate significant differences among groups (p<0.05).

Table S2 Commercial assay kits used for serum biochemical measurements and their manufacturer-reported performance characteristics

Indicator	Manufacturer	Catalogue No.	Assay method	Detection/linear range	Sensitivity/LOD	Precision
Estradiol (E2)	Cloud-Clone Corp.	CEA461Ge	Competitive inhibition ELISA	12.35-1,000 pg/mL	<4.93 pg/mL	Intra-assay CV <10%; inter-assay CV <12%
Procollagen type I N-terminal propeptide (PINP)	Cloud-Clone Corp.	CEA957Mu	Competitive inhibition ELISA	370.4-30,000 pg/mL	<138.2 pg/mL	Intra-assay CV <10%; inter-assay CV <12%
C-terminal telopeptide of type I collagen (CTX-I)	Cloud-Clone Corp.	CEA665Mu	Competitive inhibition ELISA	123.5-10,000 pg/mL	<51.3 pg/mL	Intra-assay CV <10%; inter-assay CV <12%
Bone alkaline phosphatase (BALP)	Nanjing Jiancheng Bioengineering Institute	H234-1-2	Sandwich ELISA	0-20 ng/mL	NR	CV ≤3%; inter-batch relative range ≤5%
Osteocalcin (OCN)	Cloud-Clone Corp.	SEA471Mu	Sandwich ELISA	0.156-20 ng/mL	<51.3 pg/mL	Intra-assay CV <10%; inter-assay CV <12%
Calcium (Ca)	Nanjing Jiancheng Bioengineering Institute	C004-2-1	MTB colorimetric method, microplate	0-2 mmol/L	NR	Within-batch difference ≤4%; between-batch difference ≤6%
Inorganic phosphorus (P)	Nanjing Jiancheng Bioengineering Institute	C006-1-1	Phosphomolybdic acid colorimetric method	0.02-2 mmol/L	LOD: 0.02 mmol/L	Intra-batch CV 2.3%; inter-batch CV 3.1%
Leptin	Cloud-Clone Corp.	SEA084Mu	Sandwich ELISA	0.156-10 ng/mL	<0.054 ng/mL	Intra-assay CV <10%; inter-assay CV <12%
Adiponectin (APN)	Cloud-Clone Corp.	SEA605Mu	Sandwich ELISA	0.156-10 ng/mL	<0.063 ng/mL	Intra-assay CV <10%; inter-assay CV <12%
Total cholesterol (TC)	Nanjing Jiancheng Bioengineering Institute	A111-1-1	COD-PAP method, microplate	0-19.39 mmol/L	NR	CV ≤3%; inter-batch relative range ≤5%
Triglyceride (TG)	Nanjing Jiancheng Bioengineering Institute	A110-1-1	GPO-PAP enzymatic method, microplate	0.3-11.4 mmol/L	ΔA = 0.2000-0.4000 at 2.7 mmol/L²	Measurement precision ≤5.0%; inter-batch difference ≤8.0%
High-density lipoprotein cholesterol (HDL-C)	Nanjing Jiancheng Bioengineering Institute	A112-1-1	Enzymatic colorimetric method, microplate	0.09-2.50 mmol/L	ΔA >0.04 at 1.00 mmol/L	CV ≤3%; inter-batch relative range ≤5%
Low-density lipoprotein cholesterol (LDL-C)	Nanjing Jiancheng Bioengineering Institute	A113-1-1	Enzymatic colorimetric method, microplate	0.61-18.0 mmol/L	ΔA = 0.180-0.280 at 2.6 mmol/L	CV ≤8%; inter-batch relative deviation ≤10%

Note: NR, not reported in the manufacturer documentation.

Table S3 Primer sequences used for RT-qPCR

Gene	Forward (5'-3')	Reverse (5'-3')
Gapdh	AGGTCGGTGTGAACGGATTTG	TGTAGACCATGTAGTTGAGGTCA
Pparg	GACATTCCATTCACAAGAGC	TTCAGAATAATAAGGTGGAGATGC
Cebpa	CAAGAACAGCAACGAGTACCG	GTCACTGGTCAACTCCAGCAC
Runx2	CCGCCTCAGTGATTTAGGTT	TGGGGAGGATTTGTGAAGAC
Sp7	TCGGAGAGGAGGAGGAAGAC	CAGGCTGGTAGTGGGTTGTA
Tnfsf11	CGCTGCACTACAGAAACTGG	ACAGGTCTGTGTCGAGTCC
Nfkb1	CAGGAGCTGGAGGAGAAAGA	AGGGACGTTGTTGAGGAGAG
Nfatc1	GACCCGGAGTTCGACTTCG	TGACACTAGGGGACACATAACTG
Ctsk	GAAGAAGACTCACCAGAAGCAG	TCCAGGTTATGGGCAGAGATT
Dcstamp	AAAACCCTTGGGCTGTTCTT	AATCATGGACGACTCCTTGG
Acp5	CACTCCCACCCTGAGATTTGT	CATCGTCTGCACGGTTCTG

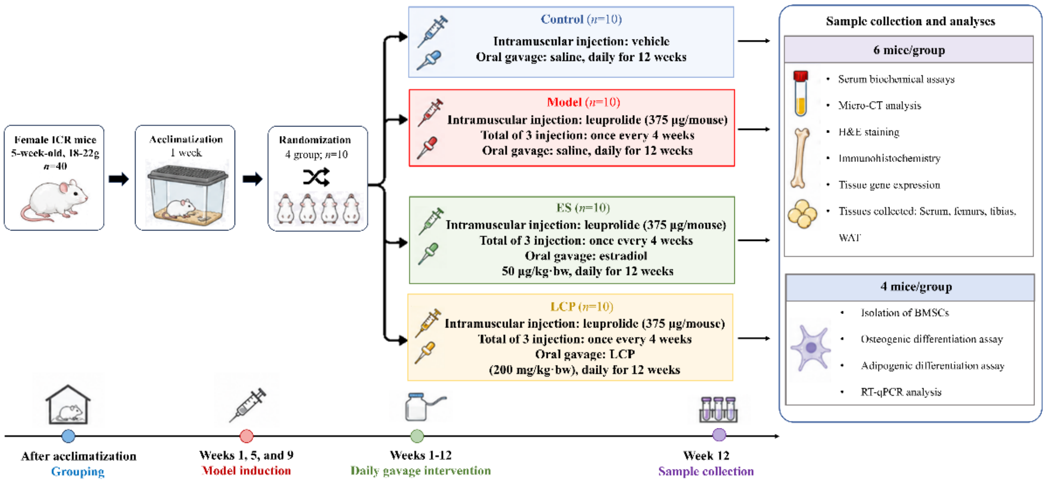

Figure S1. Schematic illustration of the experimental design in leuprorelin-induced estrogen-deficient mice. After one week of acclimatization, female ICR mice were randomly divided into four groups (*n*=10 per group): control, model, ES, and LCP. Mice in the model, ES, and LCP groups received intramuscular injections of leuprolide microspheres once every 4 weeks for a total of three injections. During the 12-week experimental period, mice in the ES and LCP groups were orally gavaged once daily with estradiol or LCP, respectively, while mice in the control and model groups received physiological saline.

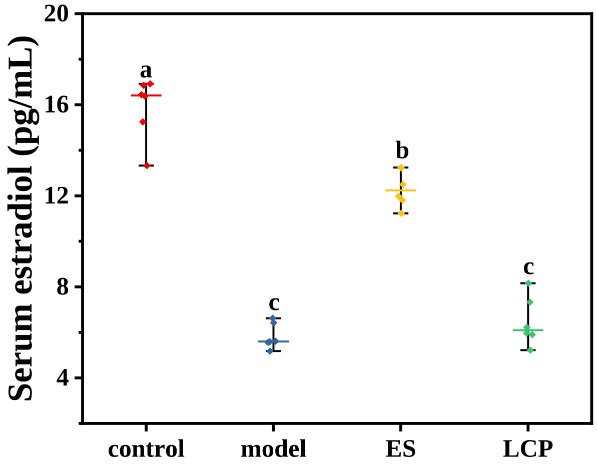

Figure S2. Serum estradiol (E2) levels in leuprorelin-induced estrogen-deficient mice. ES represents the estradiol positive-control group. Data are presented as mean ± SD (*n*=6). Different letters indicate significant differences among groups (p<0.05).

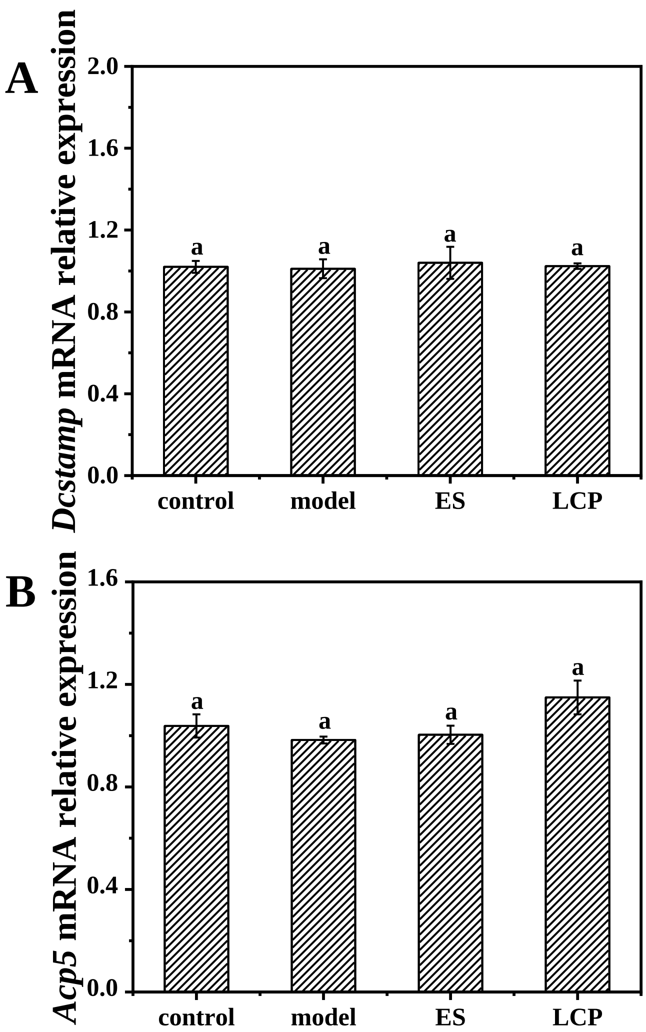

Figure S3. Effects of LCP on additional osteoclast-associated gene expression in femoral bone tissue. Relative mRNA expression levels of *Dcstamp* (A) and *Acp5* (B) were analyzed by RT-qPCR. Data are presented as mean ± SD (n=6). Different letters indicate significant differences among groups (p<0.05).
